# 17β-estradiol modifies human spermatozoa mitochondrial function in vitro

**DOI:** 10.1186/s12958-016-0186-5

**Published:** 2016-08-26

**Authors:** Malgorzata Kotwicka, Izabela Skibinska, Magdalena Jendraszak, Piotr Jedrzejczak

**Affiliations:** 1 Department of Cell Biology, Faculty of Health Sciences, Poznan University of Medical Sciences, Rokietnicka 5D, 60-806 Poznan, Poland; 2 Division of Infertility and Reproductive Endocrinology, Faculty of Medicine I, Poznan University of Medical Sciences, Polna 33, 60-535 Poznan, Poland

**Keywords:** Estrogen, Human spermatozoa, Mitochondrial membrane potential, Mitochondrial superoxide anion

## Abstract

**Background:**

It is assumed that spermatozoa are target cells for estrogens however, the mechanism of their action is not fully understood. The aim of this study was to investigate the influence of 17β-estradiol (E2) on the human spermatozoa mitochondrial function.

**Methods:**

The effects on spermatozoa of E2 at final concentrations of 10^−10^, 10^−8^ and 10^−6^ M were studied regarding the following phenomena: (1) kinetics of intracellular free calcium ions changes (using Fluo-3), (2) mitochondrial membrane potential ΔΨ_m_ (using JC-1 fluorochrome), (3) production of superoxide anion in mitochondria (using MitoSOX RED dye), (4) spermatozoa vitality (propidium iodide staining) and (5) phosphatidylserine membrane translocation (staining with annexin V marked with fluorescein).

**Results:**

E2 initiated rapid (within a few seconds) dose dependent increase of intracellular free calcium ions concentration. E2 was changing the mitochondrial membrane potential: 10^−8^ M initiated significant increase of percentage of high ΔΨ_m_ spermatozoa while the 10^−6^ M induced significant decrease of high ΔΨm cells. In spermatozoa stimulated with E2 10^−6^ M a significant increase of mitochondrial superoxide anion level was observed. 2 h incubation of spermatozoa with E2 did not alter cells vitality nor stimulated phosphatidylserine membrane translocation, for all three doses.

**Conclusions:**

17β-estradiol affected the human spermatozoa mitochondrial function. E2 in low concentration improved while in high concentration might deteriorate mitochondrial function.

## Background

It has been shown that estrogens play important role in the regulation of male reproductive system [1–3]. Previous studies revealed that human spermatozoa can be considered target cells for estrogens. Impact of estrogens comprises sperm capacitation, acrosome reaction, motility and fertilizing ability [4–7].

17β-estradiol (E2), the most powerful estrogen, affects target cells via estrogen receptors (ESRs): ESR1 and ESR1. Spermatozoa are transcriptionally inactive cells. E2 nongenomic signal transduction using intracellular secondary messengers is the only pathway possible [8, 9]. Nongenomic E2 effects are mediated via either membrane bound receptors or interaction with other membrane proteins and/or lipids [10]. Classic nuclear ESR1 and ESR2 receptors were described within cell membrane [11, 12]. However, considering absence of transmembrane domains in both ESRs, post-translational ESRs modification or ESRs binding with other membrane proteins are suggested. Kim et al. suspected that 45 and 66 kDa splicing ESRs variants, lacking the A/B domain, are capable to act as integral transmembrane proteins [13]. G protein coupled estrogen receptor (GPER) representing alternative pathway for rapid nongenomic answer was detected in human, boar and stallion spermatozoa [14, 15].

Both subtypes of classical ESRs are expressed in ejaculated spermatozoa however, the reports concerning their localization are not unanimous [14, 16, 17]. In our own unpublished study we observed strong expression of ESR1 and ESR2 in the midpiece region of human spermatozoa, supposing their presence in spermatozoal mitochondria. Our findings support the results obtained by Solakidi et al. with MitoTracker Red CMXRos mitochondrial marker usage [17]. Also, the study by Guido et al., exploiting the colloidal gold, indicated the presence of ESRs in spermatozoal mitochondria obtained from fertile men. The mitochondrial ESR2 expression was significantly stronger than that of the ESR1. Moreover, in patients with variocele, significant decrease of ESRs expression in the midpiece regions and tail or sporadically no ESRs expression were observed [7]. Assuming the ESRs are present in sperm mitochondria, their functions should be affected by estrogens.

Estrogens can have an impact on mitochondrial function, however the mechanism is not fully understood [18–20]. It is known that due to high lipid content mitochondria are reservoirs of cell estrogens [21]. It was indicated that besides passive estrogen diffusion, the mechanism of rapid estrogen transport to mitochondria is present within the cell, probably via receptor mediated endocytosis [22].

ESRs presence was revealed in somatic cells mitochondria. In most mitochondria the ESR2 seems to be the dominant receptor type even if both types were detected. Mitochondrial ESR2 mass analysis revealed various receptor isoforms [23]. The mechanism controlling ESR transport to mitochondria is poorly understood. It is suggested that in mitochondria, similarly as in the nucleus, the ESRs play the role of transcription factors [24]. It is postulated that estrogens act on mitochondria, not only by affecting the mitochondrial DNA. It was observed that they stimulate local, mitochondrial increase of free calcium ions (Ca^2+^) concentration. It was suggested that estrogens inhibit sodium dependent efflux of free calcium ions from mitochondria [25].

As a consequence of the increase of mitochondrial free calcium ions concentration, the increase of synthesis of reactive oxygen species (ROS) such as superoxide anion (O2*^−^), hydrogen peroxide (H_2_O_2_) and hydroxyl radical (OH^−^) occurs. This indicates that estrogen induced increase of mitochondrial Ca^2+^ concentration stimulates the activity of mitochondrial nitric oxide synthase (mtNOS), leading to inhibition of cytochrome c oxidase activity [26].

The aim of present study was to investigate the influence of 17β-estradiol on human spermatozoa mitochondrial function, based on the analysis of mitochondrial membrane potential changes and detection of mitochondrial superoxide anion.

## Methods

Semen of 10 normozoospermic men (according to WHO 2010 criteria) was analyzed. Material was obtained after sexual abstinence of 3–5 days. Spermatozoa with high motility were isolated by the swim-up technique. Ham’s F-10 medium was used as sperm cell extender.

Isolated cells were incubated with E2 in final concentrations of 10^−10^, 10^−8^ and 10^−6^ M. Spermatozoa stimulated by Ham’s F-10 medium were used as controls.

Spermatozoa mitochondrial membrane potential (ΔΨ_m_) was noted at 5, 10, 15, 20, 25, 30 and 120 min after exposure to E2.

Changes of Ca^2+^ level were examined throughout 400 s after exposure to E2, every 10 s.

Sperm vitality, phosphatidylserine membrane translocation and mitochondrial superoxide anion level were examined at 2 h after exposure to E2.

### Changes in intracellular free calcium ions level

Fluo-3 (Molecular Probes; Ex/Em = 488/526 nm) was used to study changes in free calcium ions level in human sperm. Spermatozoa (1 × 10^6^ cells/mL) were incubated with 4 μM Fluo-3 for 45 min at 37 °C according to the manufacturer’s protocol. For confocal microscopy, spermatozoa were immobilized in 1 % (w/v) agarose and then treated with E2. Microscopic images were used for gating single sperm cells in which fluorescence changes were recorded. Forty images were collected (every 10 s) and used to study the kinetics of intracellular free calcium ions changes. Spermatozoa were observed using LSM 510 confocal microscope (Zeiss, Jena, Germany) equipped with a Plan Apochromat 63×/1.4 Oil DIC objective. Sperm cells stimulated with Ham’s F-10 medium were used as a control for fluorescence intensity changes.

### Detection of mitochondrial membrane potential

To evaluate spermatozoa mitochondrial membrane potential (ΔΨ_m_) the 5,5′,6,6′-tetrachloro-1,1′,3,3′-tetraethylbenzimidazolocarbocyanine iodide (JC-1; Molecular Probes) was used. This is a lipophilic cationic compound that has the unique ability to label spermatozoa depending on either low or high mitochondrial potential. In the case of spermatozoa with high mitochondrial membrane potential (ΔΨ_m_ > 80–100 mV), the JC-1 forms aggregates emitting red to orange fluorescence (wavelength of 590 nm). In spermatozoa with low mitochondrial potential (ΔΨ_m_ < 80–100 mV), the JC-1 forms monomers emitting green fluorescence (wavelength of 525 to 530 nm). In both cases the excitation wavelength was 488 nm. JC-1 was diluted in DMSO (dimethyl sulfoxide) and added to cell suspension at a final concentration of 1 μM. The cells were incubated in darkness for 30 min in temperature of 37 °C. Afterwards the cell suspension was washed twice (5 min × 2400 rpm) with the use of Ham’s F-10 medium. JC-1 fluorescence emissions in spermatozoa treated with valinomycin (100 nM) was used as a control that prevents JC-1 aggregation. Valinomycin permeabilizes the mitochondrial membrane for K^+^ ions, and thus, dissipates the mitochondrial electrochemical potential.

The results were expressed as the percentage of cells exhibiting high mitochondrial membrane potential.

### Detection of mitochondrial superoxide anion

In order to estimate the amount of superoxide anion produced in spermatozoal mitochondria, we used MitoSOX Red fluorochrome (Molecular Probes). The analysis was performed according to the method described by Koppers et al. [27] with the use of confocal microscope and flow cytometer. MitoSOX Red stock solution (5 mM diluted in DMSO) was added to cell suspension (20 × 10^6^ cells per mL) to give the final concentration of 2 μM. Cells were incubated for 15 min in darkness at 37 °C and afterwards washed twice with F-10 medium (5 min at 600 × g). Microscopic observation was made under LSM 510 confocal microscope (Carl Zeiss GmbH, Germany). MitoSOX Red fluorescence was measured using flow cytometer FACSCalibur (Becton–Dickinson, USA). The results were expressed as the percentage of MitoSOX positive cells and as mean fluorescence intensity of MitoSOX positive cells.

### Sperm vitality and phosphatidylserine membrane translocation

To determine phosphatidylserine membrane translocation (PST) from the inner to the outer layer of the plasma membrane, the annexin-V labeled with fluorescein (AnV-FLUOS) (Roche Molecular Diagnostics, Darmstadt, Germany) was used. Simultaneously, to distinguish between viable and dead spermatozoa the propidium iodide (PI) staining was used, in the final concentration of 0.125 μg/L (Sigma-Aldrich, St. Louis, MO). Double staining was conducted according to manufacturer’s recommendations.

### Flow cytometry

The fluorescence signals of labeled spermatozoa were analyzed by flow cytometer FACSCalibur. 10 000 cells were examined for each experiment. The fluorescence of An-V-FLUOS and PI was excitated by argon laser (488 nm) and emission of An-V-FLUOS was measured in the FL1 channel (515–545 nm), while the red fluorescence of PI was detected in the FL3 channel (650 nm). The fluorescence of MitoSOX Red was analyzed in the FL2 channel (561–603 nm). The emission of JC-1 monomers and aggregates was measured in the FL1 channel (515–545 nm) and FL2 channel (561–603 nm), respectively. All data was collected and analyzed using CellQuest Pro software (v.5.2.1) (Becton–Dickinson).

### Statistical analysis

The analysis was performed using Statistica 10 software (StatSoft Inc., Tulsa, OK, USA). Nonparametric Kruskal-Wallis test with Dunn’s post hoc test were applied. Data were presented as mean ± SD and considered statistically significant at *P <* 0.05.

## Results

### Intracellular free calcium ions level changes

**Fig. 1:**
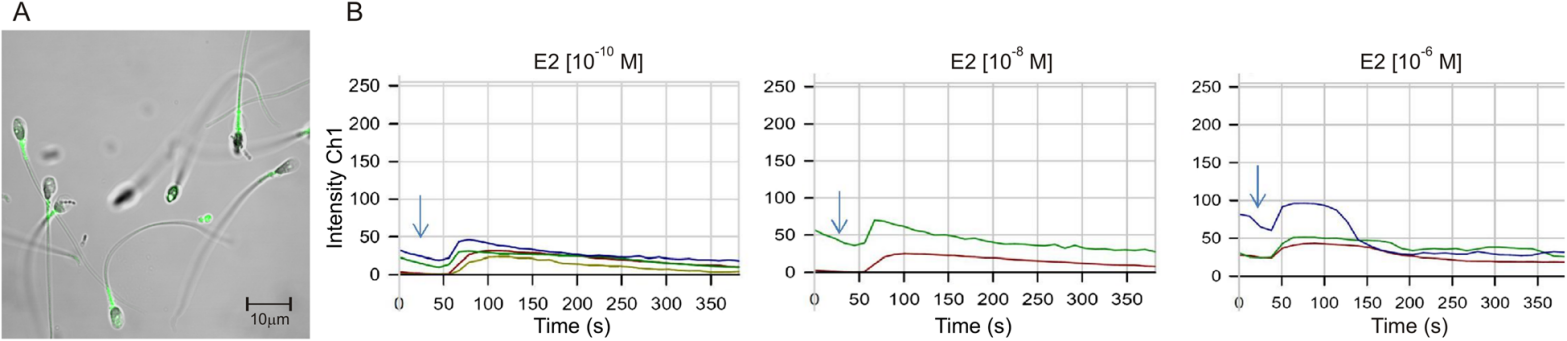
Kinetics of intracellular free calcium ions concentration changes in human spermatozoa after E2 stimulation. **a** Spermatozoa stained with Fluo-3 reveal high concentration of free calcium ions in midpiece. **b** Representative reaction after stimulation with 17β-estradiol in 10^−10^ M, 10^−8^ M or 10^−6^ M final concentration. The individual lines in the graphs correspond to the reactions of individual sperm cells. *Arrows* indicate the moment of E2 administration. Ch1-fluorescence channel for Fluo-3

In most viable spermatozoa, the highest concentration of intracellular free calcium ions was observed within the midpiece and distal part of head (Fig. 1a). E2 caused a rapid, transient increase of intracellular free calcium level. The reaction was observed at 10 s after stimulation and lasted a few minutes. The kinetics of the reaction were dose depended (Fig. 1b).

### Mitochondrial membrane potential

**Fig. 2:**
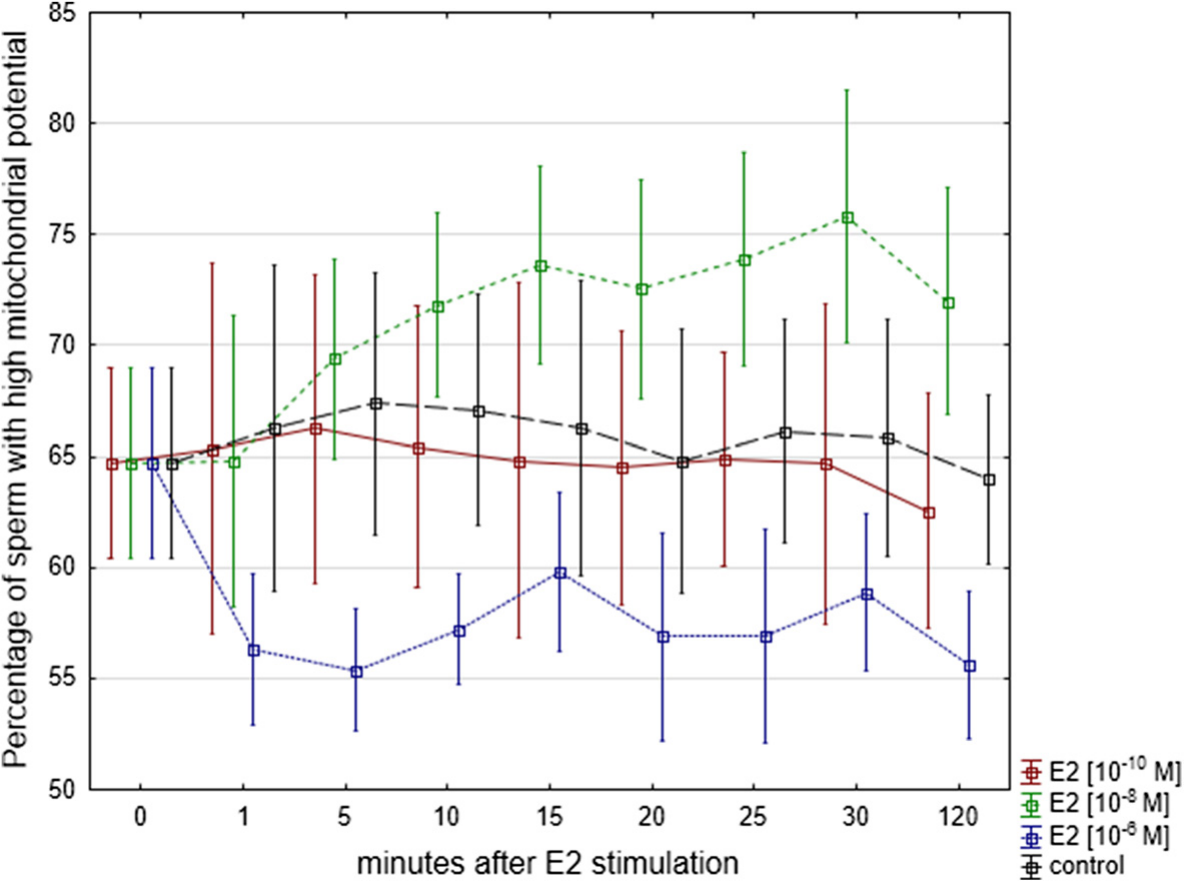
Analysis of spermatozoa mitochondrial membrane potential after exposure to E2. Kinetics of the changes of percentage of spermatozoa with high mitochondrial membrane potential measured at 1, 5, 10, 15, 20, 25, 30 and 120 min after exposure to E2 with the use of JC-1 fluorochrome. Data obtained from 10 separate analyses expressed as means ± standard deviation

**Fig. 3:**
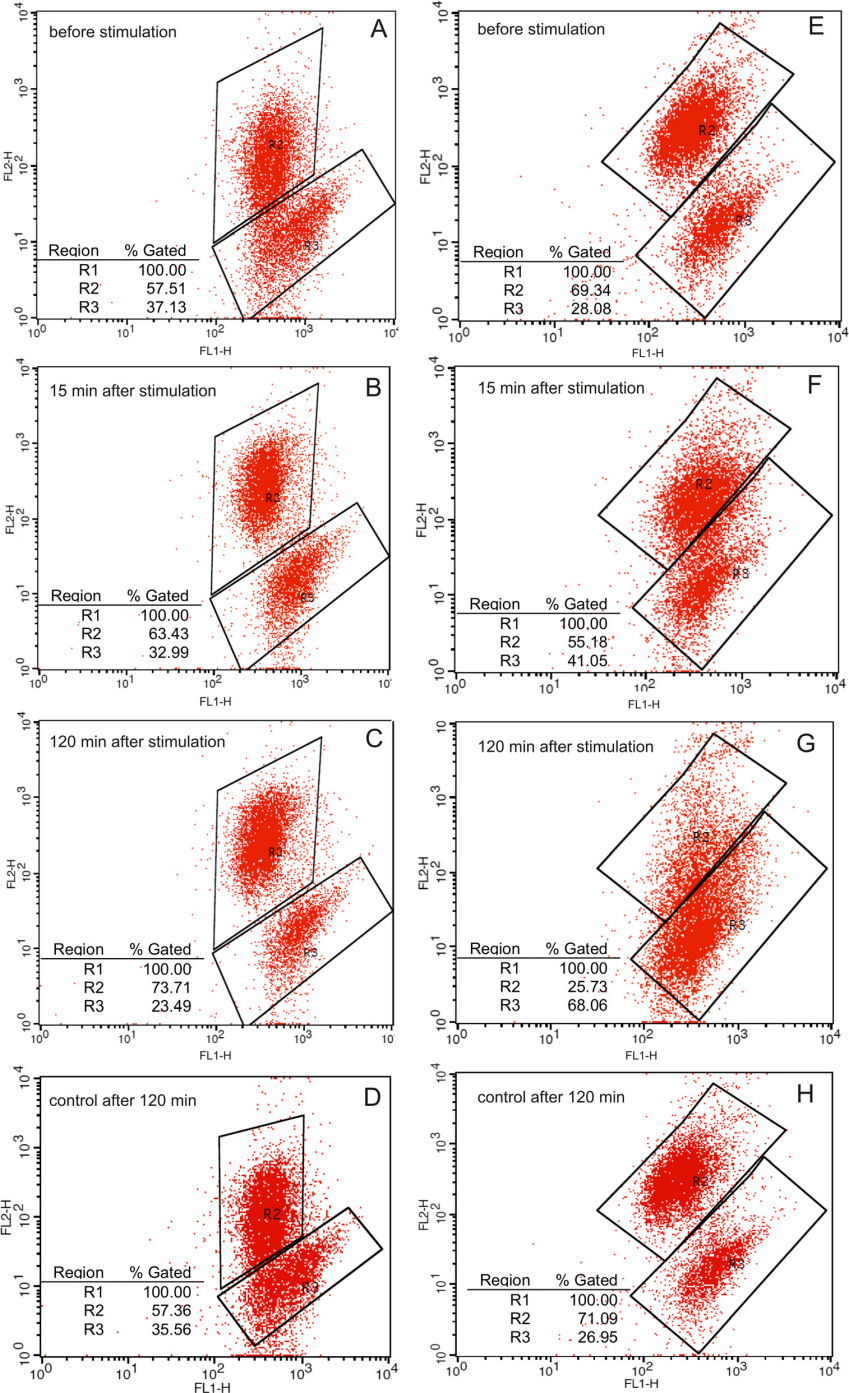
Changes of percentage of spermatozoa with high and low mitochondrial membrane potential after E2 stimulation. R2 and R3 gates represent cells with high (JC-1 aggregates emitting *red* to *orange* fluorescence) and low (JC-1 monomers emitting *green* fluorescence) mitochondrial membrane potential after E2 stimulation, respectively. Representative reactions: **a**-**c** increase of percentage of high ΔΨ spermatozoa after 10^−8^ M E2 stimulation; **e**–**g**-decrease of percentage of high ΔΨ spermatozoa after 10^−6^ M E2 stimulation. **d**, **h**-controls after 120 min stimulation

The percentage of cells with high membrane potential revealed from 60 to 92 %. No significant changes of membrane potential were observed in controls during 2 h incubation. Stimulation of human sperm cells with E2 caused significant changes of ΔΨ_m_. The 2 h incubation of sperm cells with E2 at a final concentration of 10^−10^ M did not result in statistically significant changes of ΔΨ_m_ (*P >* 0.05), while E2 at a final concentrations of 10^−8^ M and 10^−6^ M caused a significant increase (*P =* 0.004) or a significant decrease (*P =* 0.04) of the percentage of sperm cells with high ΔΨ_m_, respectively (Fig. 2 and Fig. 3). E2 induced ΔΨ_m_ changes at final concentration of 10^−8^ M were observed at 5 min while the changes at final concentration of 10^−6^ M were observed at 1 min after stimulation.

### Detection of mitochondrial superoxide anion

**Fig. 4:**
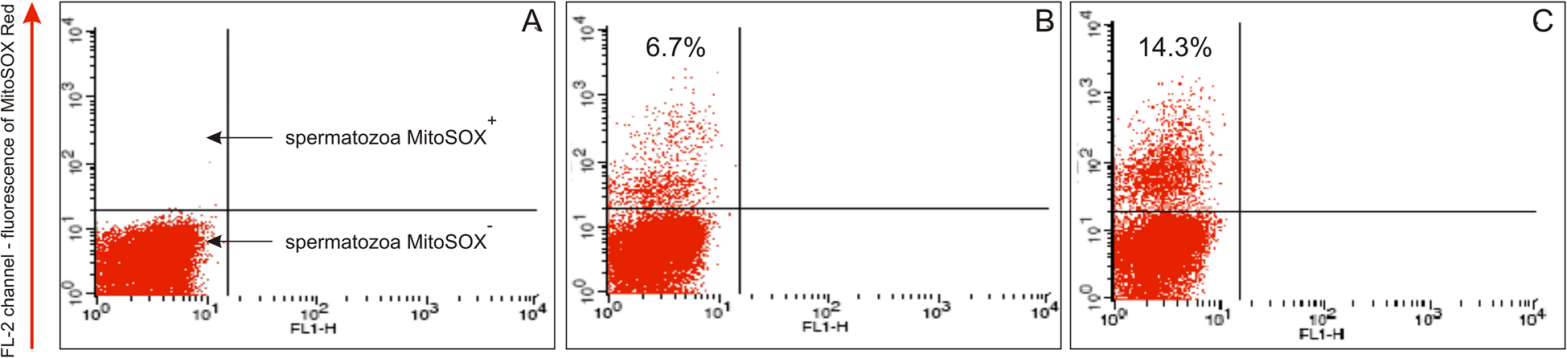
Detection of mitochondrial superoxide anion level with MitoSOX Red fluorochrome. **a** Nonlabelled cells (controls). **b**–**c** Swim-up isolated fractions of two semen samples

**Fig. 5:**
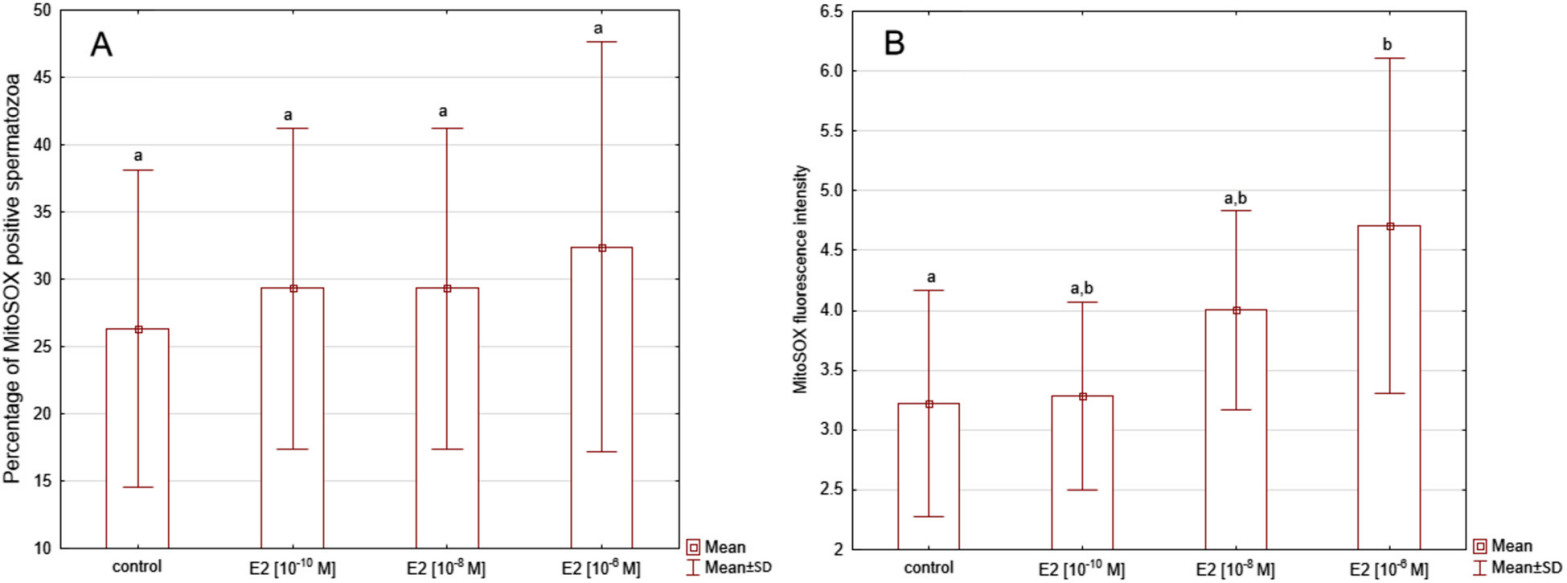
Changes of mitochondrial superoxide anion level (using MitoSOX Red dye) after 17β-estradiol stimulation. **a** Percentage of MitoSOX-positive sperm treated with E2. **b** Significant dose-dependent increase in intensity of MitoSOX fluorescence after E2 stimulation. Data obtained from 10 separate analyses are expressed as means ± standard deviation. Different superscript letters above each bar denote significant difference, *P <* 0.05

**Fig. 6:**
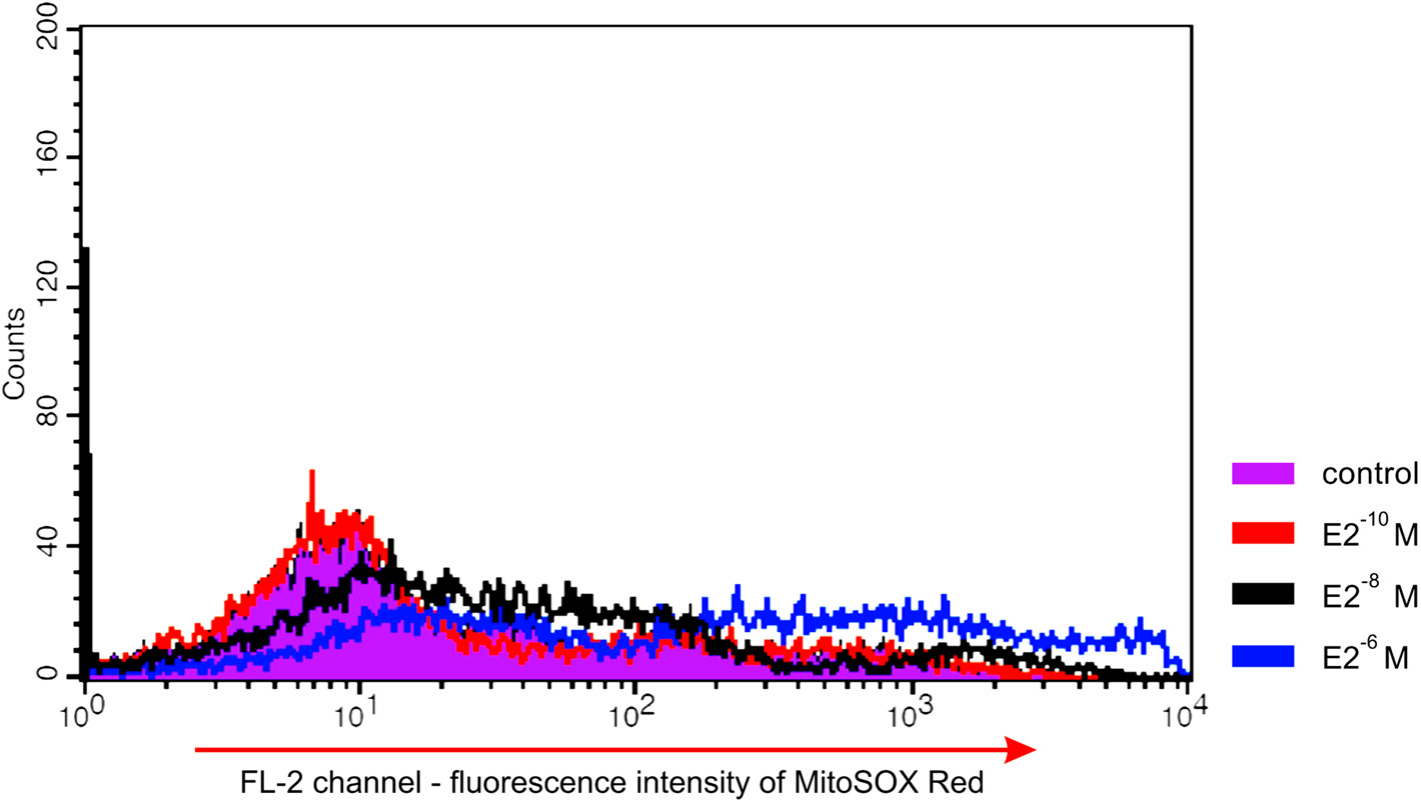
Representative examples of increase of MitoSOX fluorescence intensity after E2 stimulation. FL-2-channel of MitoSOX Red fluorescence

The swim-up isolated fraction contained two cells subpopulations: MitoSOX^−^and MitoSOX^+^ (Fig. 4). The percentage of MitoSOX positive cells ranged from 6.7 to 42 %. The 2 h incubation of sperm cells with E2 did not reveal significant change of the percentage of MitoSOX positive cells, for any of the concentrations used (*P >* 0.05) (Fig. 5a). However, a significant increase of MitoSOX fluorescence intensity was observed in cells stimulated with E2 at concentration of 10^−6^ M (*P <* 0.01) (Fig. 5b and Fig. 6).

### Effects of 17β-estradiol on sperm vitality

Flow cytometry analyses identified four fractions of spermatozoa: (1) An-V^−^/PI^−^ viable sperm without PST, (2) An-V^+^/PI^−^ viable sperm with PST, (3) An-V^−^/PI^+^ dead sperm without PST and (4) An-V^+^/PI^+^ dead sperm with PST. The sperm cells percentage of each fraction was 81.2 ± 6.1 %, 0.4 ± 0.3 %, 15.9 ± 5.5 % and 2.4 ± 1.4 %, respectively. It did not change after 2 h incubation with E2, for all concentrations used.

## Discussion

In this study the effect of E2 on mitochondrial membrane potential and mitochondrial superoxide level was examined. At the same time the effect of E2 on sperm vitality as well as changes of intracellular free calcium ions concentration were investigated.

E2 caused significant changes of mitochondrial membrane potential. When stimulating cells with E2 10^−10^ M, changes were not relevant. Concentration of 10^−8^ M caused distinct increase of percentage of high ΔΨm spermatozoa however, concentration of 10^−6^ M induced significant decrease of percentage of high ΔΨm spermatozoa. ΔΨm is an indicator of mitochondrion energetic potential. High ΔΨm denotes a functionally undisturbed mitochondrion. Positive correlation between decreased effectiveness of mitochondria and both poor sperm motility and poor capacity to fertilize the egg was described [28]. It is postulated that changes of ΔΨm can be used as an efficient tool estimating the fertilizing potential of spermatozoa devoted to in vitro fertilization [28, 29].

ΔΨm changes observed in the present study suggest that low estrogen concentration favorably influenced mitochondrial function, which, in turn, can be associated with positive impact on the vitality and motility of sperm cells. Several studies reported estrogen effects in mature spermatozoa [4–7]. Ded et al. [5] postulated significant differences in the response to tested estrogens at different capacitation time and among individual animals. It cannot be excluded that the observed calcium ions increase and the ΔΨm increase after E2 10^−10^ M stimulation are both the elements of the ongoing capacitation process. Observations regarding increase of sperm mitochondrial membrane potential under capacitation conditions have been previously made [30].

A sharp decline of ΔΨ_m_ after stimulation with higher E2 concentration appears to be an interesting finding, especially when taking into consideration the common exposure of contemporary men to xenoestrogens and their probable synergistic action with endogenous estrogens. Studies conducted on somatic cells indicate that mitochondrial estrogen’s effect largely depends on hormone dose and cell type. It was shown that estrogens influence the transportation of calcium ions to mitochondria [31, 32]. It is suggested that estrogen induced increase of free calcium ions concentration, caused by estrogens, could activate protein phosphatase which leads to dephosphorylation of cytochrome c oxidase. This, in turn, would influence the increase of membrane mitochondrial potential and production of reactive oxygen species [33]. It is proposed that estrogens can modulate ΔΨ_m_ by influencing the phosphorylation of protein complexes of the respiratory chain [33].

It can be assumed that 17β-estradiol induced changes of ΔΨ_m_ in spermatozoa could be a consequence of the influx of free calcium ions into the mitochondria. It is important to notice that the spermatozoa, in contrast to the somatic cells, present small capacities of calcium ions buffering within intracellular organelle. Calcium ions storage occurs mainly within mitochondria, as well as in acrosome and posterior part of the nucleus however, a long lasting increased calcium ions concentration may trigger pathological pathways leading to apoptosis*.* Excessive increase of mitochondrial concentration of free calcium ions is connected to the significant decrease in membrane mitochondrial potential. This, in turn, activates megachannels and in consequence causes release of cytochrome c to cytoplasm [25]. It is that estradiol induces apoptosis of rat’s spermatogenic cells by lowering the hyperpolarization of mitochondrial membrane [34].

Our study demonstrated, that sperm cells stimulated with high E2 concentrations presented decline of ΔΨ_m_ accompanied by increased mitochondrial superoxide anion level. Changes of mitochondrial ΔΨ_m_ are closely related to the issue of the oxidative stress and its influence on cell functions, the situation, when the antioxidant defensive systems fail and when the level of enzymatic and nonenzymatic molecules presenting antioxidant properties is low. It is suggested that oxidative stress is an etiological factor in various disorders, such as cardiovascular disease, neoplasms, diabetes, nervous system degenerative diseases, male and female infertility [27, 35]. To be noticed, spermatozoa are the first cells in which potential susceptibility to oxidative damages was proven. Sperm cells are very sensitive to oxidative stress mainly because of their structure. Among other, they contain polyunsaturated fatty acids (in particular docosahexaenoic acid), including six double bonds per molecule, which makes spermatozoa specific aggregates of electrons, susceptible to oxidation and other structural modifications. These modifications affect mostly cell membrane and are connected with its disturbed liquidity. This, in turn, leads to decreased motility, disturbances in acrosome reaction or in spermatozoon-oocyte fusion [35, 36]. Moreover, spermatozoa are exposed to oxidative stress because of their specific size, localization of intracellular antioxidative enzymes and limited ability of DNA repair [27, 35].

In context of the studies reporting relation between the PST and the ΔΨm decrease [37, 38] one could expect that the observed ΔΨ_m_ decline and the simultaneous increase of mitochondrial superoxide anion level in sperm stimulated with high E2 concentrations shall be accompanied with presence of sperm apoptosis markers. However, we did not observed significant increase of PST positive cells or of alive cells percentage. We speculate on the impact of the swim-up because this technique isolates spermatozoa with propoer motility and morphology as well as eliminates the spermatozoa with PST [39]. Nonapoptotic human spermatozoa with intact plasma membrane reveal the highest fertilizing potential [40]. We speculate that for this fraction the ΔΨ_m_ decrease and oxidative stress after 120 min E2 incubation did not resulted in PST. On the other hand, Grunewald et al. indicates possibility of interaction of the capacitation and apoptosis signaling systems that enables the capacitation process by prevention of apoptosis [41]. E2 involvement in capacitation could be the reason why apoptosis markers were not observed.

Both the expression of ESR in the mitochondrial location and the observation that E2 regulates the movement of spermatozoa [7] suggest that estrogens may be involved in the metabolism of mitochondria. Mitochondria take part in fundamental cell processes such as (1) cellular respiration, (2) oxidative phosphorylation, (3) apoptosis, (4) synthesis of lipids, heme, amino acids, nucleotides, steroid hormones and (5) ions homeostasis. It is suggested that mitochondria are reservoirs for estrogens. Furthermore, in mitochondria of various somatic cells both the ESR1 and ESR2 have been localized. According to up to date literature it can be assumed that estrogens take part in the regulation of mitochondrial functions. In somatic cells estrogens cause increase of mitochondrial mRNA level of mtDNA encoded proteins. That indicates that estrogens can influence the level of gene expression [18, 42].

In regular conditions mitochondria produce small amounts of ROS that can be easily neutralized by cell antioxidants. Due to low concentration the mitochondrial ROS can play role of signal molecules. Previously, estrogens were considered only as antioxidants protecting for example from neurodegenerative diseases. Some studies indicate that 17β-estradiol protects mitochondria from oxidative stress in somatic cells [43, 44]. The presented results indicate that in case of sperm cells the effect of estrogen is dependent on the hormone concentration.

It is said that the presence of estrogens may lead to increase of the level of reactive oxygen species in mitochondria by induction of nitric oxide synthase or by inhibiting cytochrome c oxidase. Also the increase of mitochondrial level of Ca^2+^ promotes ROS formation. Bennetts et al. observed that some metabolites of estradiol and xenoestrogens cause oxidative stress in human spermatozoa. An intense reaction was observed after stimulation with, for example, diethylstilbestrol or catechol estrogen derivatives. In case of genistein the effect was observed only in case of high doses of substrate used. No significant changes were observed under the influence of either 17β-estradiol, nonyphenol or bisphenol A [33].

Intracellular increase of free calcium ions level observed after E2 stimulation, suggests it is an important second messenger in nongenomic estrogen action.

## Conclusion

In conclusion, 17β-estradiol affects the human spermatozoa mitochondrial function. Results obtained in this study, in which spermatozoa were stimulated with high 17β-estradiol concentration suggest, that excessive exposition of spermatozoa on this type of may negatively affect the biology of sperm cells. This can be assumed because of observed decrease of mitochondrial membrane potential and increase of the concentration of mitochondrial superoxide anion. Consequently, it can be one of mechanisms by which the long lasting exposition of men to xenoestrogens decreases fertility potential of male gametes.

## Abbreviations

AnV-FLUOS: Annexin-V labelled with fluorescein
DMSO: Dimethyl sulfoxide
E2: 17β-estradiol
ESR1: Estrogen receptor type 1
ESR2: Estrogen receptor type 2
ESRs: Estrogen receptors
Ex/Em: Excitation/emission
Fluo-3: Fluorescence indicator of intracellular calcium
GPER: G protein coupled estrogen receptor
JC-1: 5,5′,6,6′-tetrachloro-1,1′,3,3′-tetraethylbenzimi-dazolylcarbocyanine iodide
MitoSOX*™*: Red mitochondrial superoxide indicator
mtNOS: Mitochondrial nitric oxide synthase
PI: Propidium iodide
PST: Phosphatidylserine membrane translocation
ROS: Reactive oxygen species
SD: Standard deviation
WHO: World Health Organization
ΔΨ_m_: Mitochondrial membrane potential
